# Helminth-Induced Immune Regulation: Implications for Immune Responses to Tuberculosis

**DOI:** 10.1371/journal.ppat.1004582

**Published:** 2015-01-29

**Authors:** Soumya Chatterjee, Thomas B. Nutman

**Affiliations:** Laboratory of Parasitic Diseases, National Institute of Allergy and Infectious Diseases, National Institutes of Health, Bethesda, Maryland, United States of America; University of Wisconsin Medical School, UNITED STATES

## What Is the Immunoepidemiologic Model of Helminth and Tuberculosis Coinfection?

Tuberculosis (TB) caused by the bacteria *Mycobacterium tuberculosis* (*Mtb*) remains a global cause of considerable morbidity and mortality [1]. One of the biggest current challenges of TB control is our incomplete understanding of what constitutes protective immunity in TB-endemic areas of the world. Although close to 2,200 million people maintain the infection in a state of latency and act as a reservoir of infection, the risk factors for reactivation to active disease and subsequent transmission in these populations are poorly understood. These areas also report some of the lowest rates of efficacy of the Bacillus Calmette–Guérin (BCG) vaccine [2].

Helminth infections (soil transmitted and vector-borne) exhibit broad geographic overlap with areas of TB endemicity [3]. These complex eukaryotes have the ability to establish chronic, often asymptomatic infections and have evolved highly effective methods for subverting the immune system for their survival. Their immunomodulatory effects (as we discuss below) have been shown to extend to nonparasitic infections and vaccine responses [4, 5]. Our knowledge of how helminth-coinfection-induced immune regulation can affect TB-specific immunity and disease in an endemic area comes from three broad areas of study, with specific animal models providing supplemental evidence for particular immunologic phenomena at various stages of helminth infection. First, the effects of chronic maternal helminth infection can lead to in utero sensitization to helminth antigens [6, 7] and have the potential to affect neonatal responses to BCG vaccination [8] as well as TB-specific immunity. This has important implications, as children less than 3 years of age represent the major pediatric disease burden in endemic areas [9]. Secondly, repeated exposure to vector- and soil-transmitted helminths occurs with increasing age, leading to an age-related increase both in helminth prevalence and in the rate of acquiring TB [10, 11]. Lastly, adult subjects in endemic areas with chronic helminth infection show impaired cellular responses that may alter the responses to *Mtb* antigens and possibly contribute to a higher incidence of active TB disease [12]. In this context, it is important to keep in mind some of the challenges to addressing these questions in a clinical setting. The proper diagnosis of active helminth infection can be challenging and is dependent on various factors such as the species being tested, intensity of infection [13] in a given area, and type of diagnostic test used. Also, polyparasitism is not uncommon, especially for intestinal helminth infections, and newer molecular diagnostic tests might provide higher sensitivity and specificity [14] compared to traditional stool-based techniques. Coinfection with HIV can also be an important confounder, especially for immunologic assessments in these populations. Finally, immunomodulation caused by chronic helminth infection may take a variable amount of time to resolve after treatment (depending on type of species and whether chronic sequelae are present), making prospective studies difficult to perform.

## How Does Helminth-Induced Immunomodulation Affect the Repertoire of T Cell Responses to Mycobacteria?

The question of what constitutes protective immunity in human TB is an evolving issue. A few well-defined risk factors such as advanced HIV disease and older age have been established; in addition, the pivotal protective role of a CD4+ response involving primarily interleukin 12 (IL-12), interferon gamma (IFN-γ), and tumor necrosis factor alpha (TNF-α) (Th1-like) has been established from human genetic and animal model studies [15]. There is experimental evidence that the earliest responses to the infective forms of helminth infections might actually be proinflammatory [16, 17] or of a mixed Th1/Th2 nature [18]. As patency and chronicity is established, however, there is an induction of Th2 populations as well as immunoregulatory T cell populations (both naturally occurring regulatory T cells [nTregs] and adaptive regulatory T cells [iTregs] [19, 20]). The potent immune skewing that occurs as a result of this also affects responses to bystander antigens [21]. In subjects with chronic helminth infections and evidence of mycobacterial infection, in vitro studies have revealed diminished Th1 and Th17 responses to mycobacterial antigens [22–24]; these diminished responses are related to overexpression of cytotoxic T-lymphocyte-associated protein 4 (CTLA-4), programmed cell death protein 1 (PD-1), and transforming growth factor beta (TGF-β) and to exaggerated Th2 responses [25]. Restoration of these responses has been documented after treatment of these infections [26].

## How Does the Adaptive Skewing of the Immune Response in Helminth Infections Affect Antigen-Presenting Cells (APCs)?

Studies have shown direct and indirect effects of helminths on APCs. Decreased viability and function of dendritic cells (DCs) [27] as well as down-regulation of dendritic cell-specific intercellular adhesion molecule-3-grabbing non-integrin (DC-SIGN, CD209), one of the receptors required for *Mtb* entry into DCs, was seen on exposure to live microfilariae [28]. In addition, impaired resistance to primary infection to *Mtb* was noted in a mouse model of infection with the intestinal helminth *Nippostrongylus brasiliensis* mediated through IL-4 receptor–mediated alternative macrophage activation [29]. Finally, subjects with latent TB and filarial coinfection have been shown to exhibit decreased toll-like receptor 2 (TLR2) and toll-like receptor 9 (TLR9) expression, which was reversed after successful antifilarial chemotherapy [30].

## Does Maternal Helminth Infection Affect Neonatal Immunity to TB?

It is well established from in vitro and neonatal priming studies in animals that the cytokine/chemokine milieu in which a T cell has its primary encounter with antigen determines the response (Th1/Th2) and the eventual outcome of infection [31]. It is also known that the lack of an optimal Th1 response leads to impaired immunity to mycobacterial infection [15]. Not unexpectedly, therefore, it has been demonstrated that cord blood exposure to parasite antigens from the helminth-infected mother induces both a Th2-predominant response [32] and an expansion of Tregs or IL-10-producing Type 1 regulatory (Tr1) cells. Infants who were sensitized in utero to helminth antigens exhibited a diminished or lack of IFN-γ response to the mycobacterial antigen purified protein derivative (PPD). Additionally, it was shown in the same study that a diminished IFN-γ response to PPD was noted between 10–14 months of age if the pattern of helminth antigen-induced cytokine response at birth was predominantly Th2-like. Using the diagnostic tools available to these investigators, the rates of acquisition of parasitic infection by infants enrolled in this study were very low, suggesting that helminth-induced T cell priming at birth may have long-lasting consequences for immunologic memory. The concern that antenatal parasite infection might result in impaired vaccination response to BCG [33] led eventually to a randomized double blind placebo controlled trial [34] using albendazole and/or praziquantel that demonstrated no measurable effect of maternal deworming on BCG immunization in infants at 1 year of age. Sampling bias might have affected the results of this study, as recently reported by the authors [35].

## What Is the Evidence of Helminth Infection Predisposing to TB Disease?

The association between active TB disease and coincident helminth infection has

been investigated primarily in cross-sectional studies [12], as prospective trials would need huge numbers of subjects to be followed over long periods of time. Animal models of mycobacterial challenge using different helminth species have not provided consistent results [36, 37], but it appears that helminth infection does not seem to have a significant impact on bacillary loads and clearance rates. In the only large prospective study conducted to date, baseline helminth infection status did not have any effect on incident rates of active pulmonary tuberculosis or on severity of disease [38].

## Summary and Future Directions

**Figure 1:**
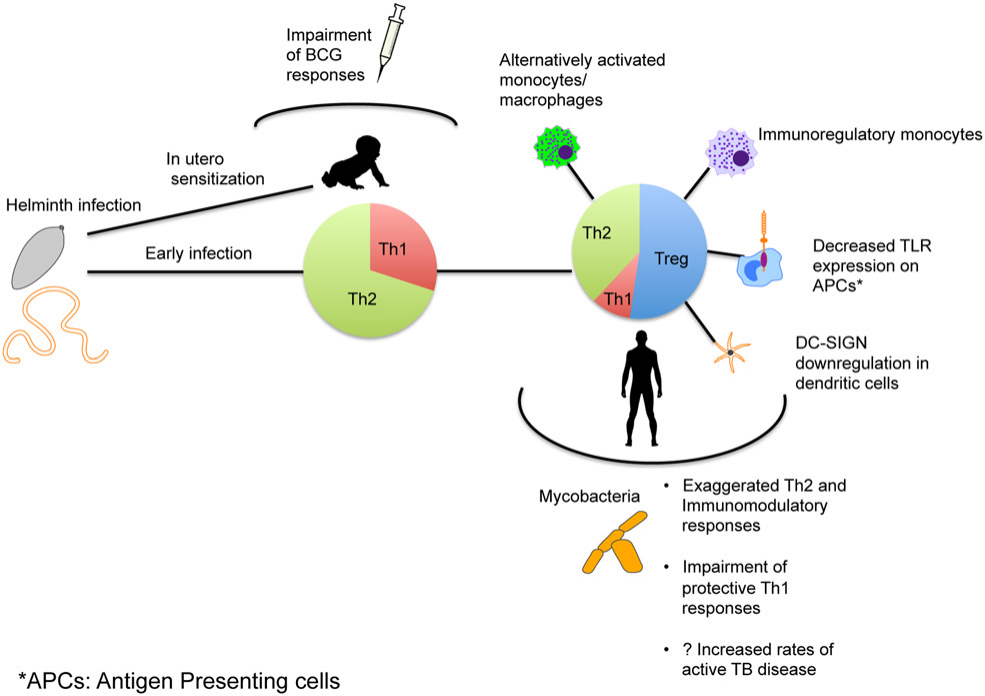
Mechanism of immune modulation caused by helminth infections affecting immune responses and susceptibility to TB. Exposure to helminth infection occurs early in areas of endemicity, with early mixed Th1/Th2 responses eventually leading to expansion of Th2 and Treg responses with establishment of chronic infection. This in turn can alter the phenotype and functionality of antigen-presenting cells as shown. The immune skewing that occurs as a result alters immune responses to *Mtb* and might affect susceptibility to TB. In utero sensitization to helminth antigens that leads to a similar skewing of the neonatal immune system can occur and thereby alter the immunogenicity of *Mtb*-specific vaccines.

Helminths have evolved complex mechanisms for immune subversion, with effects on both adaptive and innate immune responses that lead to their long-term persistence. Spillover effects on mycobacterial antigen responses have been seen in both in vitro and in vivo studies. A possible mechanism of interaction between the two infections is outlined in Fig. 1. No clear consensus has, however, emerged on whether this affects vaccine responses and enhances susceptibility to active TB disease. Although animal models provide important insights into specific pathways of immunomodulation, human studies have suffered from multiple constraints. Prospective studies following large cohorts with serial assessment for both helminth infection status as well as for development of active TB disease are logistically challenging to perform. Future studies will therefore need stringent definitions for inclusion criteria that incorporate the use of highly sensitive and specific diagnostic tools and clear enumeration of confounding variables like HIV, polyparasitism, and measures of intensity and stage of infection.
